# Reply to Teeny and Matz: Toward the robust measurement of personalized persuasion with generative AI

**DOI:** 10.1073/pnas.2418817121

**Published:** 2024-10-17

**Authors:** Kobi Hackenburg, Helen Margetts

**Affiliations:** ^a^Oxford Internet Institute, University of Oxford, Oxford OX1 2JD, United Kingdom

In our recent study, we found that using a large language model (LLM) to personalize political messages on the basis of participants’ demographic attributes did not make the messages more persuasive (1). In a response letter, Teeny and Matz caution against interpreting these results as conclusive evidence that generative AI can’t enhance personalized persuasion (2). We agree. Contrary to Teeny and Matz’s claim, we do *not* in fact conclude that personalization is an ineffective messaging strategy, or that AI cannot enhance it. In fact, we make clear that we expect other research, and future experiments, to find persuasive advantages to AI-powered personalization:

*“...we test only a single—albeit state-of-the-art—LLM and a single prompting approach. It is plausible that future, more powerful models—or different experimental approaches—may find persuasive effects of personalized messaging that we do not find here.…we might expect these findings to constitute a lower bound for the microtargeting capacities of LLMs, rather than a high-water mark.” (p. 5)*
*“...concerns around the potential misuse of AI-driven microtargeting for political influence will and should persist…” (p. 6)*

What we do conclude is that microtargeting with current LLMs is clearly not *guaranteed* to enhance persuasiveness and may in fact require more complex deployment strategies and richer data on intended targets than many may have expected.

Teeny and Matz reiterate two possible explanations for our null findings: first, that the LLM didn’t sufficiently personalize the messages. We agree; the plot Teeny and Matz present is entirely consistent with the data we report in the paper. Our own figure reporting the marginal (but nonetheless statistically significant) evidence of personalization can be found in *SI Appendix*, Fig. S11.

Second, Teeny and Matz argue that because we tested microtargeting using 10 “simple sociodemographics” instead of the “rich psychological characteristics” they use in their own research, null results were “expected.” This is a puzzling claim: decades of research in political science have demonstrated that the attributes we target (e.g., partisan affiliation, race, age, gender) play a large role in shaping political attitudes, voting behavior, and message receptivity (e.g., refs. 3–7). Furthermore, unlike psychological attributes, these sorts of characteristics are actually accessible to and commonly used by local, state, and national political campaigns, making them far more relevant for real-world political microtargeting applications (e.g., refs. 8 and 9).

In closing, we note additional strengths of our design: Unlike several of the studies Teeny and Matz cite (10, 11), our study directly measured attitude change in a randomized experiment, used a large sample, and tested targeting based on many (i.e., ref. 10) attributes. We therefore find it notable that we were unable to find a persuasive advantage to sociodemographic microtargeting using a currently top-of-class LLM. We thank Teeny and Matz for their constructive engagement with our work and look forward to future research on personalized persuasion with LLMs.
